# Discharge against medical advice from the emergency department in a university hospital

**DOI:** 10.1186/s12873-021-00422-6

**Published:** 2021-03-16

**Authors:** Feras H. Abuzeyad, Moonis Farooq, Salah Farhat Alam, Mudhaffar Ismael Ibrahim, Luma Bashmi, Shaikha Sami Aljawder, Najeh Ellouze, Abdulla Almusalam, Stephanie Hsu, Priya Das

**Affiliations:** 1 Department of Emergency Medicine, King Hamad University Hospital, Building 2345, Road 2835, Block 228, P. O. Box 24343, Busaiteen, Kingdom of Bahrain; 2 Department of Scientific Research & Development, King Hamad University Hospital, Building 2345, Road 2835, Block 228, P. O. Box 24343, Busaiteen, Kingdom of Bahrain

**Keywords:** Discharge against medical advice, Emergency medicine, Bahrain, Middle East, DAMA

## Abstract

**Background:**

Patients discharged against medical advice (DAMA) act as a high-risk population for the Emergency Department (ED), regardless of their presentations, and can pose a serious burden for the hospital. This study examines the prevalence, demographic and clinical characteristics, reasons, and clinical outcomes of a small sample of DAMA patients in a teaching university hospital, including readmission, morbidity, and mortality.

**Methods:**

A prospective, descriptive cross-sectional study was conducted in the ED of King Hamad University Hospital (KHUH) with 98,992 patient visits during a 1-year period from June 2018 to June 2019. Consenting DAMA patients were asked to complete a data collection form.

**Results:**

Patients (*n* = 413) had a mean age of 44.1 years with a female majority (57.1%). The majority were categorized as triage level-3 (87.7%). The main reasons for DAMA included refusal of the procedure/operation (23.2%), long ED waiting time (22.2%), subjective improvement with treatment (17.7%), and children at home (14.8%), whereas the least selected reason was dissatisfaction with medical care (1.2%). Follow-up of DAMA patients revealed that 86 cases (20.8%) were readmitted to the ED within 72 h of which 41 (47.7%) cases were morbidity and 2 (2.3%) were mortality. Marital status was a predictor of DAMA patients who revisit the ED within 72 h.

**Conclusion:**

The results act as a pilot study to examine a small sample of DAMA patients’ characteristics, diagnosis, and ED revisits. Hospitals should investigate further the DAMA population on a larger scale, reasons for refusing procedures, and utilize this knowledge to improve the healthcare process.

## Background

Discharge against medical advice (DAMA) can be defined as the “patient choosing to leave the hospital before the treating physician recommends discharge” [1]. DAMA is a common problem worldwide, where global prevalence rates range from 0.07 to 20% in the ED [2, 3]. DAMA can lead to negative clinical outcomes and poor healthcare resource utilization including disease relapse and high healthcare costs; it is also one of the leading causes of readmission rates [4–7]. DAMA patients are considered high-risk patient population as they experience higher mortality rates within 30 days, higher hospital readmission rates, greater length of stay in the hospital following readmission, and higher ED revisit rates of about 24% [8, 9]. Readmission rates of DAMA patients are 40% higher than patients who complete their treatment period in the hospital [6]. While treatment related costs for DAMA patients vary around the world, a study in Australia found that the cost of readmitting DAMA patients was $8.6 million per year [10]. Furthermore, other studies have reported that the hospitalization cost for DAMA patients was 50% higher than if they were fully managed and discharged by physicians during their first visit [1, 11].

In the ED, patients are categorized as DAMA if they leave before completing their care plan or while waiting to be admitted to the hospital [3, 4]. Patients who are DAMA are usually males, young, uninsured, of low socioeconomic level, with low triage acuity scale, and a history of substance abuse [3]. It is estimated that around 99% of DAMA patients have a triage level of 3, 4, or 5 according to the Manchester Triage Scale [7]. The majority of patients who sign DAMA in the ED are discharged with a diagnosis of chest pain, abdominal pain, or bronco-spastic airway disease [12].

There are various reasons for patients choosing to be DAMA in the ED, including but not limited to: family commitments, financial implications, conflict with healthcare staff, lack of medical improvement, seeking alternative traditional medicine, and long ED waiting time [3, 4]. While most studies documented patients’ reasons for DAMA, very few studies explored patient knowledge about the significance of DAMA. One particular study by Taylor and Cameron (2000) reviewed three commonly used types of discharge instruction and found that about 66% of patients were improperly informed about the harmful consequences of their decision to DAMA [13]. Their recommendations focused on the importance of proper physician-patient communication and the discharge instructions that should be provided by the ED physician to patients who sign DAMA [14].

In the Gulf Corporation Countries (GCC), only one study was found in Saudi Arabia looking at DAMA in tertiary ED patients [2]. Due to the identified gaps in the regional literature, Abuzeyad et al. conducted a study (2017) to investigate the prevalence rates and reasons for DAMA in their university hospital for all departments, where the main reasons identified for DAMA in the ED subgroup were subjective improvement (28.8%), children at home (20.4%), long waiting time (17.1%), and not agreeing to the procedure/operation (15%) [15].

The purpose of this study is to investigate the current demographic and clinical characteristics, reasons, and outcomes of DAMA patients from the ED of a public university hospital in Bahrain over a one-year period including readmission, morbidity, and mortality. Results from the study may help provide evidence on how to develop interventions to reduce DAMA rates and add to the existing literature on DAMA prevalence in the Middle East.

### Ethical considerations

The study was ethically approved by the hospital’s Institutional Review Board, which is in compliance with the National Health Regulatory Authority regarding the use of human subjects in research. Information relevant to the research study was presented in the informed consent in appropriate language. Participants were able to withdraw their participation at any stage of the research and notified that their answers would be collected anonymously. There was no financial involvement in the study.

Strict confidentiality was maintained through password protection accessible only by the listed coauthors during the collection, management, and analysis of data. According to the hospital’s Research Department guidelines, data from this study will be retained for 5 years to allow for potential follow up of the study population and will then be destroyed by permanent deletion of all data files.

## Methods

### Study design and setting

The Institutional Review Board (IRB) at KHUH approved this descriptive, cross-sectional prospective study conducted on DAMA patients in the ED. Patients who decided not to complete their treatment or who left while waiting to be admitted were asked to fill in a data collection form after signing the hospital DAMA form. This form included a list of reasons for patients choosing DAMA based on the literature, including those related to the patient, health staff, treatment, and environment. The study was conducted at the ED of KHUH, which had 98,992 patients in the period between June 2018 and June 2019. Patients were triaged using the Manchester Triage System (MTS) [16] and then were seen by an emergency physician for further management. No funding was received for this study.

### Selection of participants and recruitment

All DAMA patients during the study period were recruited through convenience sampling. According to the hospital policy for Discharge Against Medical Advice, patients that decide to leave are first seen by a nurse and then a physician. The patient is counselled by the physician regarding the patient’s concerns and reasons to sign DAMA. If the patient still prefers to leave, a hospital based DAMA form is given to the patient to sign. These were the patients who were approached to enter the study. There were a total of six researchers involved in the data collection process. Patients who agreed to take part in the study were provided with a hard copy of the informed consent, a data collection form, as well as the DAMA form. They were also informed that KHUH staff may contact them to obtain any clarifications or additional information related to the study. The researchers emphasized that all participants are free to reject participation or withdraw during the study, and that their decision would not affect the current or subsequent treatment. All patients included in the study were adults ≥18 years, competent with no evidence of an altered level of consciousness, psychiatric illness, or alcohol/drug ingestion that would impair their judgment, and could be contacted for follow up. A total of 473 DAMA patient files were included initially in the study. The files were subsequently checked 1week later to determine if these patients returned back to the ED and additional data was collected. Data was extracted from participant-filled hard copies of the data collection form, which acted as a proforma, and from patient electronic medical records.

### Measurements

Patients were presented with a hard copy data collection form by a researcher that included three sections: 1- An informed consent, 2- Participant demographics, and 3- A list of questions regarding reasons for DAMA. The informed consent included the purpose of the study, the risks and benefits, researchers’ contact details, and a notice to participants that they can withdraw from the study at any point with no repercussions. The patients’ socio-demographic variables and other secondary variables can be found in the Results section in Tables 1, 2 and 3. Researchers requested patients who fit the inclusion criteria to participate without presenting them any incentives or using coercion. Researchers explained to patients that filling in the form and participating in the study was optional, and would not have an impact on their current or future treatment at KHUH. Participants were also informed that all responses would be anonymous and confidential. Upon completion of the form, participants handed the form back to the researcher, which were placed in unlabeled folders to ensure anonymity and confidentiality of responses.

**Table 1:** Demographic data of participating patients admitted into ER who are DAMA in King Hamad University Hospital (KHUH) (*n* = 413)

Variable	Frequency (Percentage)
**Age**	
18–29	119 (28.8%)
30–39	82 (19.9%)
40–49	62 (15%)
50–59	56 (13.6%)
60–69	49 (11.8%)
70+	45 (10.9%)
**Sex**	
Male	117 (42.9%)
Female	236 (57.1%)
**Education Level**	
Illiterate	5 (1.2%)
Primary School	54 (13.1%)
Middle School	66 (16%)
High School	167 (40.4%)
University	121 (29.3%)
**Marital Status**	
Single	87 (21.1%)
Married	316 (76.5%)
Divorced	10 (2.4%)
**Living Alone**	
Yes	39 (9.4%)
No	374 (90.6%)
**Employment Status**	
Student	23 (5.6%)
Employed	179 (43.3%)
Unemployed	211 (51.1%)

**Table 2:** Admission data for DAMA patients’ visit to the ED (n = 413) at King Hamad University Hospital

Variable	Frequency (%)
**Mode of Arrival**	
Self	351 (85%)
Ambulance	48 (11.6%)
Other	14 (3.4%)
**Day of Visit**	
Sunday	44 (10.7%)
Monday	53 (12.8%)
Tuesday	45 (10.9%)
Wednesday	74 (17.9%)
Thursday	73 (17.7%)
Friday	63 (15.3%)
Saturday	61 (14.8%)
**Triage Level**	
Level 1 (Red)	0 (0)
Level 2 (Orange)	23 (5.5%)
Level 3 (Yellow)	362 (87.7%)
Level 4 (Green)	28 (6.8%)
Level 5 (Blue)	0 (0)
**Frequency of DAMA Before**	
Never	343 (83.1%)
Once	36 (8.7%)
A Few Times	28 (6.8%)
Many Times	6 (1.4%)
**Times of Presentation to ED**	
00:00–06:00	111 (26.9%)
06:01–12:00	68 (16.5%)
12:01–18:00	119 (28.8%)
18:01–00:00	115 (27.8%)
**Consulted Specialty in the ED**	
Emergency Medicine	224 (54.2%)
Internal Medicine	72 (17.4%)
Obstetrics & Gynecology	45 (10.9%)
General Surgery	40 (9.7%)
ENT	13 (3.1%)
Orthopedics	12 (2.9%)
Ophthalmology	3 (0.7%)
Cardiology	2 (0.5%)
Urology	1 (0.2%)
Maxillofacial	1 (0.2%)

**Table 3:** The list of reported diagnosis and their frequency among DAMA patients (*n* = 413)

Category of First-Visit Discharge Diagnosis	Frequency (%)
Gastrointestinal	89 (21.55%)
Cardiology	69 (16.71%)
Surgical	49 (11.86%)
Obstetrics and Gynecology	45 (10.90%)
Infectious Diseases	22 (5.33%)
Neurology	22 (5.33%)
Urology	22 (5.33%)
Trauma	21 (5.08%)
Musculoskeletal (Non-traumatic)	14 (3.39%)
Pulmonology	14 (3.39%)
Nephrology	11 (2.66%)
Hematology	9 (2.18%)
Metabolic and Endocrine	9 (2.18%)
Otorhinolaryngology	6 (1.45%)
Toxicology	6 (1.45%)
Immunology	3 (0.73%)
Ophthalmology	2 (0.48%)

### Data analysis

Statistical analyses were performed using SPSS software version 23.0 to determine the descriptive statistics (frequencies and percentages) of the DAMA patients sample. A multivariate analysis was performed using logistic regression to interpret the predictors of patient revisits after DAMA and to deduce a model, as well as explore correlations between certain demographic variables, reasons for DAMA, and revisits.

### Characteristics of study subjects

From KHUH electronic medical records, a total of 98,992 patients were seen in the ED during the period of June 2018 to June 2019, and 2464 were categorized as DAMA. From the population of DAMA patients in the ED, a total of 473 patients agreed to participate. Out of the 473 consenting DAMA patients, 60 patients (12.7%) were excluded due to incomplete data.

#### Demographic data for DAMA patients

The characteristics for the included 413 patients are shown in Table 1. The age of the patients ranged between 18 and 95 years old with a mean age of 44.1 years (standard deviation ±18.03), where the majority of DAMA patients were female (57.1%). More than 28% of DAMA patients were between 18 and 29 years old, 19.9% were 30–39 years old, 15% were 40–49 years old, 13.6% were 50–59 years old, 11.8% were 60–69 years old, and 10.9% were 70+ years old. Of the existing sample, 76.5% were married and the remaining patients were either single or divorced, only 9.4% were living alone, and 69.7% possessed a high school or university degree. In terms of employment status, 51.1% were unemployed, 43.3% were employed, and 5.6% were students.

## Results

### Admission data of DAMA patients

As seen in Table 2, the majority of DAMA patients arrived by themselves (85%) and 11.6% arrived by ambulance. Almost 70% of DAMA patients came to the ED on a weekday and 30.1% came on a weekend (in Bahrain, the weekend is on Friday and Saturday as Friday is considered a day of worship), with the most common days being Wednesday (17.9%) and Thursday (17.7%). All DAMA patients had triage levels 2, 3, or 4, with the majority of DAMA patients categorized as triage level-3 (87.7%, 362) followed by triage level-4 (6.8%, 28). It is important to note that the ED receives patients, does a triage level assessment first, and then determines the Consulted Specialty required to review that patient. The Consulted Specialty then comes to the ED to review the patient and determines the plan of management. The diagnosis is determined after the review of the ED and Consulted Specialty together.

About 17% of DAMA patients had signed a DAMA form at least once or more previously. The presenting time to ED was evenly distributed between the following three time slots: midnight and 6 am (26.9%), 12:00 pm and 6:00 pm (28.8%), and 6:00 pm to midnight (27.8%). The slowest period of time of presentation was between 6:00 am to 12:00 pm (16.5%). In terms of physician evaluation, 54.2% were seen by an emergency physician only, while 45.8% were referred to the on-call Specialist for consultation. The majority of referrals were internal medicine (17.4%), followed by obstetrics & gynecology (10.9%), and general surgery (9.7%).

### Discharge diagnosis for DAMA patients

The discharge diagnosis was analyzed per system as shown in Table 3, where the gastrointestinal system was the most common (21.6%), followed by cardiac (16.7%), surgical (11.9%), and obstetrics and gynecology (10.9%); abdominal pain followed by chest pain were the two most common symptoms.

### Reasons for DAMA

The reasons for DAMA in the ED are listed in Table 4. The most common reasons for DAMA were refusal of procedure/operation (23.2%), long ED waiting time (19.8%), subjective improvement with treatment (17.7%) and children at home (14.8%). The least common reason was dissatisfaction with medical care (1.2%). There were significant differences in reasons for DAMA between gender and age groups. Females tended to select DAMA due to children at home and refusal of procedure/operation. Men tended to choose long waiting time and subjective improvement with treatment (Chi-Square *p* < 0.005). In terms of differences between age groups, patients aged 60 years old and above tended to choose “long waiting time” and “refusal of procedure/operation” compared to the age groups of 18–39 year olds and 40–59 year olds (Chi-Square *p* = 0.042). Marital status, living alone status, employment status, and level of education had no significant impact on reasons for DAMA.

**Table 4:** List of reported reasons for leaving by DAMA patients (*n* = 413)

Reasons	Frequency (%)
Refusal of procedure / operation	99 (23.2%)
Long waiting time	92 (22.2%)
Subjective improvement with treatment	73 (17.7%)
Children at home	61 (14.8%)
Seeking another medical opinion	41 (9.9%)
Religious reasons / beliefs	12 (2.9%)
Holiday	9 (2.2%)
Dissatisfaction with medical care	5 (1.2%)
Other	21 (5.1%)

### Summary of 72-h revisit outcomes for DAMA patients

A one-week follow up of DAMA patients revealed that 86 patients **(**20.8%) had revisited the ED. All were within 72 h, of which 47.7% were then admitted to the hospital after an ED consultation. The follow-up outcomes of those patients are highlighted in Table 5. Of those patients who were admitted, the majority were diagnosed under Internal Medicine (15.1%), Obstetrics & Gynecology (9.3%), and General Surgery (8.1%). Of the 86 patients, there were 2 cases of mortality (2.3%), 18 cases (20.9%) were DAMA for the second time, and 25 cases (28.9%) were discharged home by a physician. Out of the 86 patients who presented to the ED for a second visit, 68 of them had the same diagnosis between their first and second visit. The remaining 18 patients presented with a clinical progression of the initial diagnosis.

**Table 5:** 72-h revisits outcome for DAMA patients in the ED (*n* = 86)

Outcome	Frequency (%)	Diagnosis
Total 72-h revisits	86 (20.8%)	
Total Readmission	41 (47.7%)	
Internal medicine	13 (15.1%)	Cholecystitis = 2, Stroke = 2, COPD exacerbation, Urinary Tract Infection, Severe GERD, Acute pancreatitis, Pyelonephritis, Acute hepatitis, Hyperkalemia, Hemodialysis Line Sepsis, Pneumonia
Obstetrics & Gynecology	8 (9.3%)	Complete Abortion = 5, Hyperemesis gravidarum, Labia Majora abscess, Post-Operative Intra-Abdominal Sepsis
General Surgery	7 (8.1%)	Acute appendicitis = 2, Pilonidal abscess, Sub-hepatic Abscess, Intestinal obstruction, Gluteal abscess, Neck Abscess
Urology	5 (5.8%)	Urolithiasis = 4, Hematuria for investigation
Intensive Care Unit	3 (3.5%)	End-stage renal disease with hyperkalemia, Pneumonia, Septic shock
Cardiac Care Unit	3 (3.5%)	Acute coronary syndrome = 2 (Atrial Fibrillation)
Orthopedics	1 (1.1%)	Pelvic fracture
Maxillofacial	1 (1.1%)	Mandibular fracture
**Mortality (Postmortem not performed)**	2 (2.3%)	Cardiopulmonary Arrest during emergency hemodialysis, Cardiopulmonary Arrest due to Acute Renal Failure with Hypernatremia
**DAMA patient 2nd Time**	18 (20.9%)	
**Discharged Home by Physician**	25 (28.9%)	

### Multivariate analysis

A multivariate analysis was performed using logistic regression to interpret the predictors of revisits after DAMA and to deduce a model. Variables that were entered into the model were age (≤40 and > 40), marital status (married, single and divorced), employment status (employed, unemployed, and student), triage-level and “Living alone” status, catchment area “status”. The only significant predictor of returning to the ED within 72 h included (see Table 6): Marital status (OR 0.20 95% CI (0.05–0.80)), where patients are divorced were more likely to return to the ED within 72 h. However, this significance was only at a *p*-value of 0.054.

**Table 6:** Logistic Regression Model Demonstrating Marital Status as a Predictor for DAMA patient revisits to the ED within 72 h

Variables	OR	95% CI	***P*** Value
**Age**			
≤ 40	1.064	0.622–1.82	0.820
> 40 (ref)			
**Marital Status**			
Single	0.771	0.38–1.56	0.470
Divorced	4.044	1.11–14.70	**0.034**
Married (ref)			
**Employment status**			
Employed	0.915	0.51–1.16	0.761
Student	1.180	0.35–3.92	0.787
Unemployed (ref)			
**Catchment**			
Yes	2.128	0.955–4.74	0.065
No (ref)			
**Living alone**			
Yes	0.652	0.25–1.64	0.364
No (ref)			
**Triage Level**			
Yellow (ref)			
Orange	0.745	0.23–2.37	0.619
Green	0.861	0.311–2.37	0.773

### Comparison of DAMA patients with an ED revisit after 72 h vs. DAMA patients with no revisits

Table 7 provides a summary of the comparison between DAMA patients who had revisits to the ED within 72 h versus DAMA patients who did not. There were no significant differences between the groups in terms of age, sex, “Living Alone” status, level of education, employment status, or triage level. However, there was a slightly significant difference between the two groups in terms of marital status (*p* = 0.054). Those who had a revisit within 72 h were more likely to be divorced.

**Table 7:** Between-group comparison of DAMA patients with a revisit after 72 h and DAMA patients with no revisit

	DAMA with no revisit (***n*** = 327)	DAMA with revisit (***n*** = 86)	***P***-value*
Age, mean ± SD	43. 94 ± 18.27	44.60 ± 17.26	0.55
Sex			
Female	185 (56.6%)	51 (59.3%)	0.64
Male	142 (43.4%)	35 (40.7%)	
Marital Status			
Married	250 (76.5%)	66 (76.7%)	**0.054**
Single	72 (22.0%)	15 (17.4%)	
Divorced	5 (1.5%)	5 (5.8%)	
Catchment area or Non-Catchment			
Catchment	267 (82.3%)	78 (90.7%)	0.058
Non-catchment	58 (17.7%)	8 (9.3%)	
Living alone			
Yes	33 (10.1%)	6 (7.0%)	0.379
No	294 (89.9%)	80 (93.0%)	
Level of Education			
Illiterate	5 (1.5%)	0	0.706
Primary School	41 (12.5%)	13 (15.1%)	
Middle School	53 (16.2%)	13 (15.1%)	
High School	129 (39.4%)	38 (44.2%)	
University Degree	99 (30.3%)	22 (25.6%)	
Employment status			
Employed	145 (44.3%)	34 (39.5%)	0.725
Unemployed	164 (50.2%)	47 (54.7%)	
Student	15 (5.5%)	5 (5.8%)	
Triage			
Level 2 (Orange)	19 (5.8%)	4 (4.7%)	0.836
Level 3 (Yellow)	285 (87.2%)	77 (89.5%)	
Level 4 (Green)	23 (7.0%)	5 (5.8%)	

*Bold figures indicated significant differences

## Discussion

DAMA is one of the most common issues worldwide in healthcare that leads to significant morbidity, mortality, burden on the healthcare system, and high healthcare costs. While much of the research on Emergency Medicine focuses on overcrowding in the EDs worldwide, very little attention is spent on documenting the prevalence of DAMA and its impact on patient outcomes and the healthcare system. This study aimed to determine the prevalence rate, demographic and clinical characteristics, reasons, and clinical outcomes, for DAMA in ED of a university hospital in the Kingdom of Bahrain.

All DAMA patients were categorized as triage levels-2, 3, or 4 (emergent, urgent, and less urgent, respectively), with the majority in triage level-3. Saritemur et al. (2012) found that in a university hospital setting, 99.1% of DAMA patients had triage levels-3, 4, or 5 and less than 1% had triage levels-2, however, their 5-tier triage system followed the Canadian Triage and Acuity Scale (CTAS), versus the Manchester Triage Scale, which was used in this study [6, 7]. On the other hand, El-Metwally et al. (2019) reported a higher number of DAMA patients in triage level-2 (24.5%) using CTAS, whom are categorized as emergent triage patients [2].

The most common first-visit discharge diagnosis for DAMA patients (Table 3) were related to gastrointestinal and cardiac complaints, which was consistent with other findings [3, 4, 12]. The emergency physician evaluated 54.2% patients independently, and further referrals were made to internal medicine, obstetrics & gynecology, and general surgery (17.4, 10.9, and 9.7%, respectively).

Current results reflected that DAMA patients chose similar reasons for DAMA to those from the Abuzeyad et al. (2017) study, as well as new reasons within this study [15]. Refusal of a procedure/operation accounted for 24%, while other studies reported 16.8% for refusal of an intervention [7]. It would have been informative to explore which procedures/interventions patients objected to and reasons for refusal, such as cost. These were unfortunately not measured within our study. Consequently, we have looked to literature to explore potential explanations. A study in a trilingual ED in Hong Kong [18] found that effective communication, including 1- a clear explanation of the processes and procedures including risks of DAMA, 2- a clinician’s engagement with the patient and other clinicians, and 3- contextual factors like time pressure, can influence a patient to participate in a medically advised procedure. Another study highlighted that the quantity and quality of information received by a patient can mitigate any negative emotional factors (e.g., frustration, irritation, stress, and fear), prompting patients to wait longer in the ED to get the needed medical assistance and reduce DAMA [19].

The second most common reason for DAMA was long ED waiting time (22.2%). As we were unable to measure precise waiting times for this study, we are only able to provide general context that for patients who have already been seen by an ED physician, it can take an average of 4–6 h. This can be attributed to waiting for laboratory reports, imaging reports, beds for admission, specialty consultation, a delay in the admission process, and/or a delay in treatment. In other studies, this reason accounted for 0 to 18.7% [3, 7, 20]. Our results were in line with previous findings, including those from a retrospective ED study covering a 6-year period, where the prolonged waiting time was the most common cause for DAMA [21]. The figures for other reasons, including subjective improvement from treatment, children at home, and seeking other opinions, were higher in contrast to another study [3]. About 5% of the patients chose DAMA due to religious events and holidays, especially in the holy fasting month of Ramadan and other religious holidays. Dissatisfaction with medical care had a low percentage of 1.2%, while in other institutions this can reach up to 10.3% [7]. Gender and age were the only variables that were found to have a statistically significant difference on reasons for DAMA.

Considering the risks that can accompany DAMA patients after leaving the hospital, we followed up with our patients one-week later to examine those who revisited the ED. Out of 413 patients, 86 (20.8%) had returned to our ED within the first 72-h. Admission involved 41 patients to different specialties, of which 6 were admitted to the intensive and cardiac care units. Another 18 patients decided to sign DAMA for the second time and leave the ED. Individuals whose symptoms subsided were then discharged home, accounting for 15 cases, and 10 patients were discharged with organized follow up with the outpatient clinics or the health care center. Unfortunately, there were 2 patients who presented to the ED with cardiopulmonary arrest and had unsuccessful resuscitation. As observed from the broad range of returning presentations, DAMA patients are exposing themselves to significant multiple morbidities and mortality, and are not protected regardless of the disease type. DAMA patients are among the subgroups who are expected to return back to the ED within 72 h [22], and in one study, they accounted for up to 49% of patients due to continuing or worsening of the disease process [23]. The majority of the presenting symptoms were related to the gastrointestinal and cardiovascular systems [24]. Furthermore, a comparison between DAMA patients who had a revisit within 72 h and those who did not revealed that marital status was a predictor for their return though it was not very statistically significant (*p* = 0.054). Divorced patients were more likely to revisit the hospital than single patients. One explanation could be that divorced patients could be dealing with more insecurities related to their health as they might be living alone with no one to take care of them. This would be an interesting research question to further explore in a new study.

ED healthcare performance can be evaluated by several key performance indicators for healthcare quality including ED length of stay, left without being seen, 72-h revisits, morbidity and mortality, and DAMA [17]. DAMA patients who leave during their clinical management cause a considerable dilemma for ED physicians. In this one-year study, 413 patients were included for the final data analysis, demonstrating that DAMA patients are taking a risky decision due to incomplete or interrupted treatment. We measured a concerning prevalence of morbidity and mortality in our results but due to the small sample size, we could not demonstrate that DAMA patients are at a higher risk of morbidity and mortality. We hope to perform a future cohort study that is case matched because based on literature, DAMA patients will eventually require a higher level of care with an increased cost mainly due to the considerable morbidity and mortality represented by complications and adverse events [1, 25, 26].

Understanding the risk factors and predictors will eventually improve the ED care process, mitigate complications, and reduce the operational cost. The context of multiple socioeconomic factors of the studied population, the healthcare system, the entitlement for treatment, and the alternative options should be considered when exploring this topic. Furthermore, because of the heterogeneity of the DAMA patient population and their diverse reasons for doing so, it has been suggested that such patients must be offered a high-quality DAMA which includes: justifying the need for admission, the risks of discharge, capacity determination, and providing an alternative follow-up plan [26]. The literature has emphasized the importance of documenting DAMA patients in detail through their medical charts by the emergency physician. Information documented should include the patient’s capacity, risks related to the discharge, and the DAMA form signed by the patient [6]. In fact, some have advocated obtaining informed consent, evaluating the decision-making capacity, and assessing the health literacy for any patients who are DAMA with appropriate follow-up if possible [27]. In particular, we recommend having a patient relation manager or social worker within the ED of our hospital to help communicate with patients wanting to be DAMA.

### Limitations

The study had significant limitations related to the small sample size, setting, and method of collecting data. The first limitation was that the study was conducted in one center only, which is not representative and thus cannot be generalized to the rest of the Bahraini ED population. Second, only 20% of DAMA patients participated in our study, which means that there is a high likelihood that study participants were not representative of all DAMA patients at KHUH and differ substantially from the overall ED and DAMA cohorts. Another limitation was related to the inability to follow up with DAMA patients who did not return to our ED, and whose symptoms might have subsided, worsened, presented to another institution.

## Conclusion

The reasons for DAMA can vary from one ED to another depending on characteristics of the population, culture, the ED system within a hospital, and country regulations. We encountered significant limitations during the study that limit our ability to conclude concrete associations with all DAMA patients in Bahrain. Our results rather provide increased insight into a sample of DAMA patients seen at our hospital during the timeframe of this study. Our study showed the dominance of certain reasons for DAMA in the ED, including refusal of the procedure/treatment and that ED wait times were too long. We established in our patient sample that over 20% returned to the ED within 72-h. The logistic regression modelling showed that marital status was a predictor of DAMA patient revisits. Based on the concerning potential of these results, the refusal of medical care can impose an independent risk factor to the patients, and emergency physicians must discuss and emphasize the potential risks of DAMA to their patients. Furthermore, this provides implications for healthcare professionals to develop larger scale studies examining the prevalence, characteristics and reasons for DAMA, which can lend to informing interventions that bring awareness of the risks of DAMA and reduce its likelihood, by tailoring it according to gender, age groups, and marital status. A future multi-centric study inclusive of all DAMA patients as a proportion of all ED patients will help to better capture its occurrence in Bahrain.

## Data Availability

The datasets used and/or analysed during the current study are available from the corresponding author on reasonable request. All data generated or analysed during this study are included in this published article.

## Abbreviations

CTAS: Canadian Triage and Acuity Scale
DAMA: Discharge against medical advice
ED: Emergency Department
GCC: Gulf Corporation Countries
IRB: Institutional Review Board
KHUH: King Hamad University Hospital
MTS: Manchester Triage System
